# Total ankle arthroplasty versus ankle arthrodesis—a comparison of outcomes over the last decade

**DOI:** 10.1186/s13018-017-0576-1

**Published:** 2017-05-18

**Authors:** Cort D. Lawton, Bennet A. Butler, Robert G. Dekker, Adam Prescott, Anish R. Kadakia

**Affiliations:** 1 Department of Orthopedic Surgery, Northwestern University, Chicago, IL 60661 USA; 2 Department of Orthopedic Surgery, Feinberg School of Medicine, Foot and Ankle Orthopedic Fellowship, Northwestern University – Northwestern Memorial Hospital, Chicago, IL USA; 3 676 North Saint Clair, Suite 1350, Chicago, IL 60611 USA; 4 259 East Erie, 13th Floor, Chicago, IL 60611 USA

**Keywords:** Total ankle arthroplasty, Ankle arthrodesis, Tibiotalar arthritis, Ankle arthritis

## Abstract

**Background:**

The surgical treatment of end-stage tibiotalar arthritis continues to be a controversial topic. Advances in surgical technique and implant design have lead to improved outcomes after both ankle arthrodesis (AA) and total ankle arthroplasty (TAA), yet a clear consensus regarding the most ideal form of treatment is lacking. In this study, the outcomes and complications following AA and TAA are compared in order to improve our understanding and decision-making for care and treatment of symptomatic tibiotalar arthritis.

**Methods:**

Studies reporting on outcomes and complications following TAA or AA were obtained for review from the PubMed database between January 2006 and July 2016. Results from studies reporting on a minimum of 200 total ankle arthroplasties or a minimum of 80 ankle arthrodesis procedures were reviewed and pooled for analysis. All studies directly comparing outcomes and complications between TAA and AA were also included for review. Only studies including modern third-generation TAA implants approved for use in the USA (HINTEGRA, STAR, Salto, INBONE) were included.

**Results:**

A total of six studies reporting on outcomes following TAA and five reporting on outcomes following AA met inclusion criteria and were included for pooled data analysis. The adjusted overall complication rate was higher for AA (26.9%) compared to TAA (19.7%), with similar findings in the non-revision reoperation rate (12.9% for AA compared to 9.5% for TAA). The adjusted revision reoperation rate for TAA (7.9%) was higher than AA (5.4%). Analysis of results from ten studies directly comparing TAA to AA suggests a more symmetric gait and less impairment on uneven surfaces after TAA.

**Conclusions:**

Pooled data analysis demonstrated a higher overall complication rate after AA, but a higher reoperation rate for revision after TAA. Based on the existing literature, the decision to proceed with TAA or AA for end-stage ankle arthritis should be made on an individual patient basis.

## Background

End-stage arthritis of the tibiotalar joint is a disabling condition that causes substantial functional impairment and decreased quality of life [1]. The most common etiology is post-traumatic arthritis, with other causes including rheumatoid arthritis, idiopathic arthritis, neuropathic arthritis, osteonecrosis, hemophilic arthritis, septic arthritis, and gout [2]. Total ankle arthroplasty (TAA) and ankle arthrodesis (AA) are the two primary surgical options for patients who fail conservative measures.

Ankle arthrodesis has long been considered the gold standard for treatment of end-stage arthritis. Critics of arthrodesis, however, cite high complication rates and altered function as reasons to justify alternative forms of joint-sparing treatment [3]. For example, nonunion rates as high as 43% in some high-risk sub-groups have been reported after ankle arthrodesis [4]. There is also concern that elimination of tibiotalar motion accelerates adjacent joint degeneration due to the loss of a major motion segment [5, 6]. Still, despite successful arthrodesis, loss of normal ankle motion has been shown to negatively affect functional status at long-term follow-up [7]. Greater attention to soft tissue management and newer fixation options has resulted in fewer complications, higher fusion rates, and more predictable pain relief [8–12].

Total ankle arthroplasty was developed as an alternative to ankle arthrodesis. While first-generation implants were fraught with unacceptably high complication rates, current third-generation designs have led to more favorable outcomes [13–19]. Still, long-term survivorship of third-generation implants remains unknown. Proponents of TAA believe the advent of current generation designs and refined surgical techniques have the potential to offer long-term outcomes equivalent to that of arthrodesis, with benefits including preservation of ankle motion, improved gait, and preservation of adjacent joints.

The majority of existing studies comparing AA to TAA show equivocal or conflicting results. In addition, many of these studies focus on older techniques and early generation implants, which further complicate interpretation of the literature. The purpose of this study is to compare the outcomes of AA and TAA using pooled results from studies published in the past 10 years that include only modern third-generation TAA implants.

## Methods

### Search strategy

**Fig. 1:**
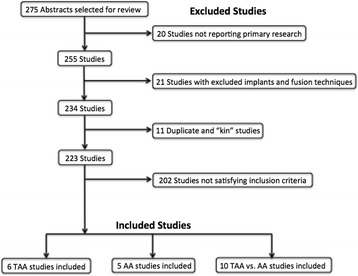
Decision flow chart for included studies. *Abbreviations*: *TAA* total ankle arthroplasty, *AA* ankle arthrodesis, *kin studies* studies reporting results from the same patient population at different time intervals

An electronic search for publications available in the English language from January 2006 to July 2016 using the PubMed database was performed. The keywords used included “ankle arthritis,” “tibiotalar,” “ankle arthroplasty,” and “ankle arthrodesis.” Two independent authors (CL and BB) reviewed all abstracts from the PubMed search results. This was supplemented with a manual review of references in all review articles and primary full-text studies identified in the PubMed database to verify the inclusion of all relevant publications. Our literature search decision flow chart for study inclusion is depicted in Fig. 1.

### Total ankle arthroplasty study selection

Eligibility for study inclusion from the PubMed database search was assessed independently by two different authors (CL and BB). The review sought to identify primary research reporting outcomes and complications from third-generation total ankle arthroplasty designs approved for use in the USA (HINTEGRA, STAR, Salto, INBONE). Articles with a minimum of 200 ankles reporting complication, reoperation, and/or revision data were included for analysis in this review. Exclusion criteria included studies published prior to January 2006; abstracts, review articles, and surgical technique articles which did not report primary research outcomes; use of implants other than one of the four third-generation implants listed above; publications reporting only on restricted patient cohorts selected from a larger and more generalizable population; and studies reporting on revision TAA (Table 1).

**Table 1:** Study inclusion and exclusion criteria

Study type	Inclusion criteria	Exclusion criteria
Total ankle arthroplasty studies	Published since January 2006 Third-generation implant approved for use in the USA Specific implants: HINTEGRA, STAR, Salto, INBONE Minimum of 200 ankles Report primary research Report complication, reoperation, and/or revision rate data	Revision data Duplicate data Non-consecutive series Restricted patient cohorts
Ankle arthrodesis studies	Published since January 2006 Open and/or arthroscopic fusion technique Minimum of 80 ankles Report primary research Report complication, reoperation, and/or revision rate data	Revision data Duplicate data Non-consecutive series Restricted patient cohorts
Total ankle arthroplasty vs. arthrodesis studies	Published since January 2006 Meet implant and fusion technique criteria stated above Report primary research	Revision data Duplicate data Non-consecutive series Restricted patient cohorts

Abbreviations: *TAA* total ankle arthroplasty, *AA* ankle arthrodesis, *kin studies* studies reporting results from the same patient population at different time intervals

### Ankle arthrodesis study selection

A similar identification and screening strategy was used to analyze the PubMed database search results for ankle arthrodesis publications. The review sought to identify primary research reporting outcomes and complications for ankle arthrodesis. Studies with a minimum of 80 ankles reporting complication, reoperation, and/or revision data were included for analysis in this review. A cutoff of 80 ankles was chosen to identify studies reporting on larger cohorts with a goal of identifying a similar number of articles to use for comparison to the TAA articles in pooled data analysis. Exclusion criteria included studies published prior to January 2006; abstracts, review articles, and surgical technique articles which do not report primary research outcomes; articles reporting only on restricted patient cohorts selected from a larger and more generalizable population; and studies reporting solely on fusion accomplished with external fixation techniques or revision AA (Table 1).

### Total ankle arthroplasty versus ankle arthrodesis study selection

A similar identification and screening strategy was used to analyze the PubMed database search results for publications reporting data directly comparing TAA to AA. The review sought to identify primary research reporting data directly comparing an AA cohort to a cohort of patients treated with a third-generation total ankle arthroplasty design approved for use in the USA. There was no cutoff for minimum number of ankles or data reported for inclusion in this review to provide a more comprehensive analysis of the literature directly comparing the two treatment options. Exclusion criteria included studies published prior to January 2006; abstracts, review articles, and surgical technique articles which do not report primary research outcomes; use of implants other than one of the four third-generation implants described above; and studies reporting on revision TAA or AA (Table 1).

### Data collection and analysis

The total ankle arthroplasty and ankle arthrodesis studies included for analysis in this review can be seen in Table 1. Demographic information including study design, recruitment period, number of ankles, TAA prosthesis or AA operative technique, mean follow-up, and mean patient age was collected. The totals and adjusted means were then reported. Adjusted means were calculated for mean follow-up and mean age in the TAA and AA studies by summing each studies’ mean follow-up or mean age multiplied by the number of ankles in their study and dividing this sum by the total number of ankles in all studies reporting the variable of interest.

Complications, non-revision reoperations, and revisions were recorded for each study as seen in Table 2. There was significant heterogeneity between studies with respect to reporting complications. When a complication of interest, non-revision reoperation rate, or revision rate was not explicitly stated within an article, attempts were made to calculate these rates using data reported in the study. The studies reviewed did not consistently report on all of the same types of complications. Therefore, an adjusted rate was calculated by taking the number of specific complications divided by the sum of all cases for only those studies reporting the outcome of interest. Perioperative and postoperative fractures were combined in our analysis and reported as an overall fracture rate. During interpretation of revision rates, revision of TAA was defined as removal of either the tibial or talar component or both components with subsequent placement of an antibiotic spacer, reimplantation of metal components, conversion to an arthrodesis, or amputation. Revision of AA was defined as return to the operating room for a revision fusion in the setting of a nonunion.

**Table 2:** Demographic information of total ankle arthroplasty and ankle arthrodesis studies

Author	Year published	Study design	Recruitment period	Number of ankles	Prosthesis (TAA)/operative technique (AA)	Mean follow-up in years (range)	Mean age in years (range)
Total ankle arthroplasty (TAA)							
Gross et al. [14]	2016	Prospective	2007–2013	455	INBONE (219)/STAR (151)/Salto (85)	3.7	62.0
Demetracopoulos et al. [15]	2015	Prospective	2007–2011	395	INBONE (214)/STAR (104)/Salto (77)	3.5 (2–5.4)	63.1
Lewis et al. [16]	2015	Prospective	2007–2012	249	INBONE	3.3	63.2
Barg et al. [17]	2013	Retrospective	2000–2010	722	HINTEGRA	6.3 ± 2.9 (2–12.2)	61.1 ± 12.6 (19.8–90.0)
Schenk et al. [18]	2011	Prospective	2001–2007	218	Salto	3.5	56.8 ± 11.2
Wood et al. [19]	2008	Prospective	1993–2000	200	STAR	7.3 (5–13)	59.6 (18–83)
Total/adjusted mean	2008–2016	Prospective (5) Retrospective (1)	1993–2013	2239	INBONE (682) STAR (455) Salto (380) HINTEGRA (722)	4.8 years	61.3 years
Ankle arthrodesis (AA)							
Chalayon et al. [8]	2015	Retrospective	2002–2013	215	Open	NR	56 ± 14 (18–88)
Gordon et al. [9]	2013	Retrospective	2004–2009	82	Open	3.9 (0.58–8.3)	56.1 (18–75)
Zwipp et al. [10]	2010	Retrospective	1994–2000	94	Open	5.9 (4.8–7.8)	53 (34–69)
Nielsen et al. [11]	2008	Retrospective	1994–2005	107	Open (49)/arthroscopic (58)	NR	51.92 (20–84)
Muckley et al. [12]	2007	Retrospective	1993–2001	137	Open	3.5 (1–7.5)	49 (21–79)
Total/adjusted mean	2007–2015	Retrospective (5)	1993–2013	635	Open (577) Arthroscopic (58)	4.3 years	53.4 years

Abbreviations: *TAA* total ankle arthroplasty, *AA* ankle arthrodesis, *NR* not reported

Studies comparing TAA to AA included for analysis in this review can be seen in Table 3. Demographic information was collected including study design, number of ankles, TAA prosthesis or AA operative technique, mean and/or minimum follow-up, outcome measures used, and major study conclusions.

**Table 3:** Total ankle arthroplasty and ankle arthrodesis study complications, reoperations, and failures

		Complication rate							
Author	Number of ankles	Wound complications (*n*)	Fracture (*n*)	Deep infection (*n*)	TAA aseptic loosening/AA nonunion (*n*)	Overall rate (*n*)	Non-revision reoperation rate	Revision for implant failure (TAA) or revision fusion (AA)	Kaplan-Meier survivorship analysis
Total ankle arthroplasty (TAA)									
Gross et al. [14]	455	NR	NR	1.5% (7)	NR	14.1% (64)	NR	3.1% (14)	NR
Demetracopoulos et al. [15]	395	6.6% (26)	NR	0.8% (3)	NR	NR	9.6% (38)	5.1% (20)	NR
Lewis et al. [16]	249	8.4% (21)	3.6% (9)	1.2% (3)	4.0% (10)	NR	14.5% (36)	*8.4%* (*21*)	*NR*
Barg et al. [17]	722	NR	NR	0.4% (3)	5.8% (42)	NR	NR	8.4% (61)	94.0% at 5 years 84.0% at 10 years
Schenk et al. [18]	218	2.3% (5)	2.3% (5)	1.4% (3)	1.8% (4)	NR	NR	16.5% (36)	86.6% at 5 years (95% CI 81.2–91.9)
Wood et al. [19]	200	2.5% (5)	9.5% (19)	NR	12.5% (25)	32.5% (65)	3.0% (6)	12.0% (24)	93.3% at 5 years (95% CI 89.8–96.8) 80.3% at 10 years (95% CI 71.0–89.6)
Adjusted rate	2239	5.4% (57/1062)	4.9% (33/667)	0.9% (19/2039)	5.8% (81/1389)	19.7% (129/655)	9.5% (80/844)	7.9% (*176*/2239)	
Ankle arthrodesis (AA)									
Chalayon et al. [8]	215	14.4% (31)	0.9% (2)	5.1% (11)	9.3% (20)	40.9% (88)	11.2% (24)	7.4% (16)	NR
Gordon et al. [9]	82	2.4% (2)	1.2% (1)	0% (0)	0% (0)	20.7% (17)	14.6% (12)	0% (0)	NR
Zwipp et al. [10]	94	5.3% (5)	0% (0)	0% (0)	1.1% (1)	9.6% (9)	3.2% (3)	1.1% (1)	NR
Nielsen et al. [11]	107	NR	NR	2.8% (3)	10.3% (11)	NR	23.4% (25)	4.7% (5)	NR
Muckley et al. [12]	110	10% (11)	0.9% (1)	7.3% (8)	14.5% (16)	19.1% (21)	NR	10% (11)	NR
Adjusted rate	608	9.8% (49/501)	0.8% (4/501)	3.6% (22/608)	7.9% (48/608)	26.9% (135/501)	12.9% (64/498)	5.4% (33/608)	

Abbreviations: *TAA* total ankle arthroplasty, *AA* ankle arthrodesis, *NR* not reported, *CI* confidence interval

## Results

### Total ankle arthroplasty

There were six studies that met the inclusion criteria for pooled data analysis of outcomes after total ankle arthroplasty (Table 2) [14–19]. Five of the studies were prospective and one was retrospective. The studies report on a total of 2239 ankles operated on from 1993 to 2013. INBONE was used in 682 ankles, STAR in 455 ankles, Salto in 380 ankles, and HINTEGRA in 722 ankles. The adjusted mean follow-up was 4.8 years (range 3.3–7.3 years). The mean patient age at the time of arthroplasty was 61.3 years (range 56.8–63.2 years).

### Ankle arthrodesis

A total of five studies met the inclusion criteria for pooled data analysis of outcomes after ankle arthrodesis (Table 2) [8–12]. All studies were retrospective in nature. The studies report on a total of 635 ankles operated on from 1993 to 2013. Arthrodesis was preformed through an open approach in 577 ankles and through an arthroscopic approach in 58 ankles. Three of the studies reported mean follow-up with an adjusted mean follow-up of 4.3 years (range 3.5–5.9 years). The mean patient age at the time of arthrodesis was 53.4 years (range 49–56.1 years).

### Complications, reoperations, and revisions

Table 3 lists the complication, reoperation, and revision rates in the selected studies. The most frequently reported complication in the arthroplasty group was aseptic loosening (5.8%), followed by wound complications (5.4%), fracture (4.9%), and deep infection (0.9%). Patients who underwent arthrodesis experienced mainly wound complications (9.8%), followed by nonunion (7.9%), deep infection (3.6%), and fracture (0.8%). Overall complication rate was reported in two arthroplasty studies with a mean rate of 19.7% compared to a mean of 26.9% reported in four arthrodesis studies. The pooled mean non-revision reoperation rate was higher in the arthrodesis studies (12.9%) compared to the arthroplasty studies (9.5%). Revision rate was higher in the arthroplasty group (7.9%) compared to the arthrodesis group (5.4%). Kaplan-Meier survivorship analysis was reported in four of the arthroplasty studies as seen in Table 3.

### Total ankle arthroplasty versus ankle arthrodesis

There were ten studies that met the inclusion criteria for direct comparison of total ankle arthroplasty to ankle arthrodesis (Table 4) [20–29]. Three studies were prospective and seven were retrospective. The number of ankles included in the studies ranged from 36 to 672 ankles. The total ankle prosthesis and arthrodesis techniques used for each study are listed in Table 4.

**Table 4:** Studies comparing total ankle arthroplasty to ankle arthrodesis

Author	Year	Study design	Number of ankles	Prosthesis (TAA) or operative technique (AA)	Mean/minimum follow-up (years)	Outcomes	Study conclusions
Braito et al. [20]	2014	Retrospective	141	TAA: HINTEGRA (101) AA: open (40)	TAA 4.2 (SD 2.03) AA 3.4 (SD 3.67) Minimum 6 months	Gait analysis AOFAS FAOS VAS Radiographic	No difference in cadence, stride length, or walking velocity No difference in mean global AOFAS No difference in adjacent joint arthritis
Chopra et al. [21]	2014	Retrospective	36	TAA: Salto (12) AA: NR (12) Control: (12)	Minimum 2 years	Gait analysis	Altered bilateral gait mechanics in AA patients Relatively fully recovered bilateral gait mechanics in TAA patients
Flavin et al. [22]	2013	Prospective	42	TAA: STAR (14) AA: NR (14) Control: (14)	Minimum 1 year	Gait analysis	No evidence of consistently superior gait function in TAA compared to AA
Jastifer et al. [23]	2014	Prospective	77	TAA: STAR (61) AA: open (16)	Minimum 1 year	VAS Buechel-Pappas score AOFAS Patient satisfaction Functional outcomes	TAA with improved Buechel-Pappas scale (*P* = 0.036) and AOFAS (*P* = 0.03) over AA TAA with improved walking upstairs (*P* = 0.013), downstairs (*P* = 0.012), and uphill (*P* = 0.016) over AA
Piriou et al. [24]	2008	Retrospective	36	TAA: Salto (12) AA: open (12) Control: (12)	Minimum 1 year	Gait analysis	AA with faster gait (*P* = 0.03), longer step length (*P* = 0.015) compared to replacement TAA with more symmetrical timing of gait (*P* = 0.015) and restored ground reaction force pattern over AA
Rouhani et al. [25]	2012	Retrospective	45	TAA: Salto (11) AA: open (9) Ankle osteoarthritis (15) Control: (10)	TAA 22 months (13–47 months) AA 33 months (6–58 months)	Gait analysis AOFAS FFI	Significant improvements in foot mobility after TAR during 50 months gait Significant impairments remained in patients with AA during 50 months gait
Saltzman et al. [26]	2009	Prospective	672	First phase TAA: STAR (158) AA: NR (66) Second phase TAA: STAR (448)	Up to 24 months	Complications Buechel-Pappas score	Significantly more wound complications (*P* = 0.011) and major complications (*P* = 0.045) and secondary surgical intervention in pivotal study TAA group over pivotal study AA group Significantly greater efficacy (*P* < 0.001) and overall success (*P* < 0.001) in pivotal STAR over AA The hypothesis of non-inferiority of ankle replacement was met for overall patient success
Saltzman et al. [27]	2010	Retrospective	71	TAA: STAR (42) AA: (29)	TAA 3.8 (2.2–4.3) AA 4.8 (2.2–5.9)	Complications SF-36 AOS Radiographic	Improved outcome in TAA over AA for SF-36 MCS (*P* = 0.011) and AOS pain scale (*P* = 0.001)
Schuh et al. [28]	2012	Retrospective	41	TAA: HINTEGRA (20) AA: open (21)	TAA 39.0 months (SD 17.0 months) AA 30.0 months (SD 22.0 months)	AOFAS UCLA score Satisfaction Functional outcomes	No significant differences between the groups concerning activity levels, participation in sports activities, UCLA, and AOFAS score
Singer et al. [29]	2013	Retrospective	44	TAA: (17) AA: (17) Control: (10)	TAA 1.27 (SD 0.62) AA 1.60 (SD 0.65)	Gait analysis	Gait patterns more closely resemble normal gait for TAA cohort over AA cohort

Abbreviations: *TAA* total ankle arthroplasty, *AA* ankle arthrodesis, *NR* not reported, *SD* standard deviation

Gait analysis was compared in seven studies. Two reported no significant difference between the TAA and AA cohorts [20, 22]. The remaining five studies found that patients performed better on gait analysis after TAA [21, 23–25, 29]. Clinical outcomes were collected in six studies [20, 23, 25–28]. Jastifer et al. reported improved Beuchel-Pappas scale (*p* = 0.036) and AOFAS (*p* = 0.03) in the TAA cohort over the AA cohort [23]. Saltzman et al. reported significantly greater efficacy (*p* < 0.001) and overall success (*p* < 0.001) in the Pivotal STAR group over the AA group [26]. Saltzman et al. reported improved outcome in TAA over AA for SF-36 MCS (*p* = 0.011) and AOS-pain scale (*p* = 0.001) [27]. The remaining three studies reported no significant difference in clinical outcomes between study cohorts. Two studies reported on radiographic outcomes showing no significant difference between TAA and AA in adjacent joint arthritis at the final follow-up [20, 27].

## Discussion

End-stage arthritis of the tibiotalar joint is a disabling condition resulting in decreased functionality and quality of life [1]. Ankle arthrodesis has traditionally been considered the gold standard surgical option for tibiotalar arthritis, but total ankle arthroplasty is emerging as a viable alternative for certain patient cohorts. Over the past few decades, improved surgical techniques in AA and advancements in TAA prosthesis design have led to improved outcomes with both procedures. Many of the existing studies reporting outcomes following AA and TAA focus on outdated techniques and older generation TAA implants. This review focused on outcomes following AA and TAA with modern techniques and third-generation TAA implants.

Analysis of pooled data revealed that the overall complication rate of AA (26.9%) was higher than that of TAA (19.7%), as seen in Table 3. Issues with wound healing were the most common complication reported after AA (9.8%), followed by nonunion (7.9%) and deep infection (3.6%). The adjusted rate of deep infection after TAA was 0.9%, with fractures being the most infrequent complication seen in the AA cohort at 0.8%. The most frequent complication in the TAA studies was aseptic loosening with an adjusted rate of 5.8%, followed by wound complications (5.4%) and fracture (4.9%).

The adjusted non-revision reoperation rate was also higher for AA (12.9 versus 9.5%). Chalayon et al. reported on 215 ankle fusions with the most common reason for non-revision reoperation being hardware removal (6%) followed by incision and drainage for presumed infection (5%) [8]. Nielsen et al. reported on 107 ankle fusions and demonstrated reoperation rates similar to Chalayon et al., with hardware removal (21%) and incision and drainage for deep infection (3%) as the most common reasons for non-revision reoperation [11]. The two TAA studies with the highest non-revision reoperation rates did not provide detailed analysis of their indications for reoperation [15, 16].

The adjusted revision rate, however, was higher for TAA (7.9%) compared to AA (5.4%). In their study, Wood et al. reported a 12% revision rate for a cohort of 200 total ankles with a mean follow-up of 7.3 years. The majority of patients required revision for aseptic loosening (7%) [19]. Chalayon et al. reported a 7.4% revision rate in an AA cohort of 215 ankles, primarily for nonunion [8].

Studies directly comparing TAA to AA reported a wide range of outcome measures. Seven studies comparing the two treatments examined gait analysis. Common findings included improved walking ability upstairs, downstairs, and on uneven surfaces. Most notably, patients who received a TAA demonstrated a more symmetric gait compared to those who underwent AA.

Only two studies examined adjacent joint degeneration after surgical treatment. Braito et al. discussed radiographic findings in 101 TAA patients and 40 AA patients concluding an increase in degeneration of adjacent joints following both TAA and AA with no significant difference between the two cohorts at a mean observation period of 4.2 years in the TAA group and 3.4 years in the AA group [20]. Saltzman et al. found similar results on radiographic analysis, concluding no significant difference in the change of adjacent joint arthritis between the TAA and AA cohorts [27].

Arthroscopic ankle arthrodesis has the advantage of achieving fusion with less soft tissue dissection than what is required with open arthrodesis techniques, with the literature reporting promising initial outcome data. Myerson and Quill reported a retrospective study comparing open and arthroscopic arthrodesis concluding a shorter time to fusion in the arthroscopic group [30]. O’Brien et al. reported a retrospective study demonstrating similar fusion rates with less morbidity, shorter operative times, and shorter hospital stays with arthroscopic fusion [31]. Arthroscopic ankle arthrodesis has gained increasing popularity over the past decade [32, 33]. In a recent study, Duan et al. reported a wound complication rate of 1.5% in 68 arthroscopic ankle fusions, which is much less than the adjusted wound complication seen in this study [34]. As arthroscopic fusion techniques continue to develop, the potential for improved fusion rates with fewer complications in fusion cohorts may be seen; however, more large quality outcome studies are needed.

Terrell et al. assessed practice patterns in TAA and AA in the USA, reporting an increase in the percent of TAA cases performed between 2004 and 2009 [35]. The growing popularity of TAA combined with the higher failure rates, especially at long-term follow-up, are resulting in an increasing number of TAA patients requiring revision fusion procedure. Salvage fusion for failed TAA presents challenges of bone loss and poor soft tissue envelope quality when compared to primary fusion patients. As a result, salvage arthrodesis for failed TAA has shown worse outcomes over those seen after primary fusion [36, 37]. This should be considered when deciding between TAA and AA for the treatment of tibiotalar arthritis.

Previously published systematic reviews comparing results following TAA to AA are limited, largely focusing on clinical outcomes and revision rates. Jordan et al. concluded that a few studies report functional improvement following TAA over AA; however, the lack of high-quality evidence limits a definitive conclusion on which treatment is superior [38]. Haddad et al. report a 7% revision rate following TAA compared to 9% following AA [39]. van Heiningen et al. found a similar revision rate with TAA compared to AA (11 versus 12%) in rheumatoid arthritis patients, concluding both interventions show clinical improvement, with neither procedure showing superior outcomes [40]. Our current review focuses on modern techniques and implant designs to more accurately compare the current status of these treatment options.

The current literature does not support significant advantages of one procedure over the other in the general population. Knowledge of each procedure perioperative complication profile is important to help guide treatment recommendations, which should be made on a case-by-case basis. Appropriate patient selection plays a key role in successful treatment, where one method might be preferred over the other for specific case conditions. Krause et al. suggest age less than 50 years old, arthritis secondary to trauma or neuromuscular disease, unilateral ankle arthritis, ankle range of motion less than 10 degrees, absence of arthritis in adjacent joints, severe ankle instability, and coronal deformity greater than 15 degrees as major criteria to consider and suggest AA is the favored treatment in such cases [41].

The authors acknowledge that the present study is not without limitation. Foremost, this study was limited by the lack of randomized controlled trials published in the current literature on outcomes after TAA and AA. In addition, existing studies present significant clinical heterogeneity making comparison difficult. It should be noted that our ankle fusion studies included both open and arthroscopic techniques. Studies have reported improved fusion rates and lower complication rates with arthroscopic fusion; however, this was not a focus in our current study and therefore was not separated in our analysis of complications, reoperations, and revision for the fusion studies. Further, variations existed pertaining to the manner in which outcomes, complications, and revisions were reported. Various complications were inconsistently reported, with definitions of non-revision reoperation and implant failure differing between studies. Lastly, differences in length of follow-up and lack of consistent long-term follow-up likely underestimate the true complication and failure rates reported.

## Conclusions

Total ankle arthroplasty and ankle arthrodesis are two surgical treatment options for end-stage tibiotalar arthritis supported in the literature. Currently, there is a lack of high-quality randomized controlled trials comparing these treatments in their modern form, utilizing current techniques and implant designs. The cohort studies and case series identified by this review were difficult to interpret as a whole due to heterogeneous populations and inconsistent reporting of complications and outcomes. However, pooled analysis of the data suggests that although AA may have a higher total complication rate, TAA may have a higher revision rate. Therefore, until a greater degree of current data is available demonstrating a significant advantage between the two treatment options, the decision to proceed with TAA or AA should be made on a case-by-case basis, accounting for appropriate patient selection, discussions regarding pros and cons of each treatment choice, and knowledge of perioperative complication profiles with each procedure. Individual patient goals, expectations, and understanding of the differences between the respective treatment options are vital to guide the decision between treatment with TAA or AA.

## Abbreviations

AA: Ankle arthrodesis
TAA: Total ankle arthroplasty
